# Association between the Lipid Levels and Single Nucleotide Polymorphisms of *ABCA1*, *APOE* and *HMGCR* Genes in Subjects with Spontaneous Preterm Delivery

**DOI:** 10.1371/journal.pone.0135785

**Published:** 2015-08-24

**Authors:** Lin Li, Jin Hua, Huang Jian-Ping, Long Yan

**Affiliations:** Department of Gynecology & Obstetrics, Beijing Friendship Hospital, Capital Medical University, 95 Yong’an Road, Xuanwu District, Beijing, 100050, China; Xavier Bichat Medical School, INSERM-CNRS - Université Paris Diderot, FRANCE

## Abstract

Spontaneous preterm delivery (SPTD) with gestational age between 28 and 37 complete weeks was reported to have a genetic predisposition in lipids metabolism. This study aimed to investigate the association between the lipid levels and gene polymorphisms of *ABCA1* (rs2422493), *APOE* (rs7412) and *HMGCR* (rs12916) in Chinese pregnant women with SPTD. A case-control study was conducted at the baseline randomization in 200 SPTD and 178 healthy full term delivery (FTD) women. Maternal blood lipids were detected close to delivery of fetus in SPTD group and in FTD group with gestational age-matched. Cord blood lipids were detected after delivery in two groups. Three genotypes both in maternal and cord blood were determined by real time PCR. The results showed that the levels of total cholesterol (TCHO), triglyceride (TG), high density lipoprotein (HDL), and low-density lipoprotein cholesterol (LDL) in the maternal blood in the SPTD group were significantly lower than those in the FTD group, while the levels of TCHO, HDL, and LDL in the cord blood in the SPTD group were significantly higher than those in the FTD group. In the SPTD subjects, the levels of TG and LDL in the maternal blood were associated with different genotypes of *HMGCR* gene rs12916 loci. These results indicate that abnormal lipid metabolism may exist in SPTD women and the premature fetus and the *HMGCR* gene may be a susceptible gene for SPTD.

## Introduction

Preterm delivery (PTD) is the leading cause of prenatal morbidity and mortality. The incidence of PTD is approximately 7.5–12.5% worldwide [1]. The probabilities of having cardiovascular disease and other adverse consequences are higher in PTD babies than those in full-term delivery (FTD) babies [2]. Despite many efforts that have been made by researchers and doctors, the incidence of preterm birth has not been significantly decreased.

The major clinical subtypes of preterm delivery include iatrogenic (medically indicated) preterm delivery and spontaneous preterm delivery. Intrauterine infection and premature rupture of membranes are the common causes for SPTD, there are still one half patients without any clear reason [3]. Epidemiological studies have demonstrated that PTD has a genetic predisposition [4]. Previous studies [5,6] have mainly focused on the genes and proteins that are related to infection. But the genes that contribute to prenatal nutrition status have not been extensively investigated. The abnormal change of lipid profiles in pregnant women is a risk factors for PTD [7]. Fetal serum cholesterol and lipoprotein concentrations differ between preterm and term born neonates [8]. Thus, we hypothesized that the variation of genes involved in lipid metabolism both in mother and fetus may regulate the level of lipid profiles, which contributing to the occurrence of SPTD. Here we focus on SPTD instead of PTD to exclude iatrogenic preterm delivery, which were not clarified in most previous studies.

The *ABCA1*, *APOE* and *HMGCR* genes play a critical role in lipid metabolism related to premature coronary artery disease [9–11]. However, little is known about the correlation between the polymorphisms of these genes and SPTD. The *ABCA1* gene, a member of the ATP-binding cassette A (ABCA1) transporter superfamily, encodes a membrane protein that facilitates the cellular efflux of cholesterol and phospholipids. The 477C/T single nucleotide polymorphism (SNP) in the *ABCA1* gene promoter region is a factor that affects plasma HDL level in the Chinese Han population [9]. APOE protein, encoded by *APOE* gene, plays an important role in the transport, storage, utilization and excretion of lipid. The frequency of this gene among various races and regions is different, and is closely related with hyperlipidemia, atherosclerosis and coronary heart disease [10]. The human *HMGCR* gene encodes the β-hydroxy-β-methylglutaryl coenzyme. A reductase is the rate-controlling enzyme of the mevalonate pathway that produces cholesterol and other isoprenoids. In a pharmacokinetic study [11], the polymorphism of the rs12916 locus in the *HMGCR* gene was found in a Chinese population, and a decreased low-density lipoprotein cholesterol (LDL) level was found to be related to the rs12916 locus in the *HMGCR* gene.

Little was known about the contribution of variants in fetal and maternal cholesterol metabolism genes *ABCA1*, *APOE* and *HMGCR* in SPTD. Thus, in this study we aimed to examine the lipid levels in the mother and fetus and to investigate the association between the lipid levels and polymorphisms of three major genes in Chinese SPTD patients.

## Materials and Methods

### Subjects

SPTD women with gestational age between 28 and 37 complete weeks and FTD control subjects (delivery ≥37 weeks) were recruited from the Department of Obstetrics at Beijing Friendship Hospital, Capital Medical University, and Beijing Maternal and Child Health Hospital in Haidian District between January 2011 and April 2012. Written consent was obtained from all subjects. This study was approved by the ethics committee of Capital Medical University. All included subjects met the following criteria: 21–35 years old, non-smoking, non-vegetarian, confirmed single live birth by ultrasound, and body mass index (BMI) of 19–25 kg/m^2^. The subjects with existing infection, pregnancy complications, adverse pregnancy history and iatrogenic preterm delivery were excluded from this study. Neonates of singleton pregnancies with birth weight inadequate for gestational age (lower than the 10th percentile and larger than the 90th percentile), fetal anomalies, abnormal fetal karyotype, and positive TORCH screening results were excluded.

### Detection of blood lipids

In SPTD subjects, 3.5 ml of venous blood were collected close to delivery of fetus, while in the FTD subjects, the same volume of venous blood were collected between 28^+0^ and 36^+6^ gestational week matched with SPTD subjects. Three and one-half milliliter of umbilical arterial blood in two groups were collected after delivery. Blood samples were sent to the Clinical Laboratory Center in Beijing Friendship Hospital. The levels of TCHO, HDL, LDL and TG were measured with an AU2700 automatic biochemical analyzer (OLIMPUS, Toyko, Japan).

### Detection of gene polymorphisms in the maternal blood and umbilical cord blood

The blood cells from maternal venous blood and umbilical arterial blood were separated by centrifugation at 3,000 rpm for 20 min. DNA was extracted with a genomic DNA extraction kit (TIANGEN Beijing Co., Ltd., Beijing, China). The primers and probes were designed and synthesized by Applied Biosystems (Life Technologies, Grand Islands, NY). Two probes were designed for each SNP with VIC or FAM dye labels. The probes and primers of three SNP loci were mixed as a 40× stock solution (40× TaqMan SNP Genotyping Assay Mix). The details of the three loci are shown in Table 1. The PCR reaction system was carried out in a 10μl volume containing 10μl TaqMan Universal PCR Master Mix (2×), 0.5μl primers and probes mixture (20×), and 4.5μl DNA (concentration was more than 10 ng/μl). The amplification reaction was performed at 95°C for 10 min, 50 cycles of denaturation at 92°C for 15 s and annealing/extension at 60°C for 1 min on a 7500 real-time fluorescence PCR system (Applied Biosystems). All SNPs were re-genotyped by direct sequencing in a subset of 120 individuals to check for genotyping discrepancies. Genotyping error rates were ≤0.01 for all SNPs.

**Table 1:** The basic characteristic of all SNP for ABCA1, APOE and HMGCR.

Gene SNP	Chromosomal location	Assay Id.	Nucleotide exchange	Localization	HWE *P*
*ABCA1* rs2422493	9q31.1d	C_16235101_10	GCCTAGGCTGGGGTGAGGGGAAGGC[T/C]GACAGTCCTCTGGGTAATGGGCTGC	promoter	0.1
*APOE* rs7412	19q13.32a	C_904973_10	CCGCGATGCCGATGACCTGCAGAAG[C/T]GCCTGGCAGTGTACCAGGCCGGGGC	Exon non-syn	0.18
*HMGCR* rs12916	5q13.3b	C_7445046_10	CAGTGCAATTGACCTTCTCCCTCAC[C/T]CCTGCCAGTTGAAAATGGATTTTTA	3ˊUTR	0.22

### Statistical analysis

The chi-squared test was used to determine whether the observed genotype frequencies were compatible with the Hardy-Weinberg equilibrium (HWE) and to test for differences in genotype frequencies between preterm women and term women. The data such as age, BMI, parity, and gestation week were presented as mean ± SD for continuous variables. The differences of dominant or recessive allele between two groups were analyzed with an unpaired t test. The comparison of blood lipids with different genotypes was carried out with analysis of variance (ANOVA). The Bonferroni post hoc test correction was used to correct for multiple comparisons. A generalized linear model (GLM) was used to analyze the relationships between the SNPs and lipids adjusted for age, BMI and gestation of delivery, which were identified as significant variables from the stepwise linear regression model. SPSS 12.0 software (Chicago, IL) was used to perform the statistical analysis. Each SNP were tested separately. All tests were two-side and significance level was set at 0.05.

## Results

### Demographic characteristics and lipid levels of the study population

The SPTD subjects (n = 200) had an average age of 28.83 ± 3.22 years and a BMI of 21.64 ± 3.02 kg/m^2^, while the FTD subjects (n = 178) had an average age of 28.85 ± 3.12 years and a BMI of 21.27 ± 2.82 kg/m^2^. The characteristics and lipid levels of the subjects were presented in Table 2. There were no significant differences in age, parity and BMI between SPTD and FTD groups. A generalized linear model (GLM) analysis also showed that there were no relationships between the SNPs and lipids adjusted for age, BMI and gestation of delivery.

**Table 2:** Characteristics of the study population (n = 378).

	Total(n = 378)	Preterm (n = 200)	Term(n = 178)	*P*
Age (years)	28.84 ± 3.16	28.83 ± 3.22	28.85 ± 3.12	0.934
Parity	1.5 ±0.24	1.5 ±0.25	1.5 ±0.24	0.550
BMI (Kg/m^2^)	21.42±4.78	22.32 ± 4.30	21.03 ±5.20	0.719
Gestation of delivery	36.70±2.89	34.44± 1.99	39.23± 1.09	0.000
TCHO^a^	6.26± 1.22	6.12± 1.31	6.40± 1.12	0.027
TG^a^	3.19± 1.20	3.04± 1.17	3.34± 1.21	0.019
HDL^a^	1.82± 0.39	1.77± 0.41	1.88±0.36	0.006
LDL^a^	3.56± 0.76	3.46± 0.80	3.65± 0.72	0.016
TCHO^b^	1.89± 0.56	2.05±0.58	1.65±0.44	0.000
TG^b^	0.29± 0.15	0.28±0.16	0.30±0.14	0.372
HDL^b^	0.76± 0.17	0.78±0.17	0.73±0.18	0.043
LDL^b^	0.93± 0.34	1.02±0.34	0.77±0.27	0.000

^a^ Lipid levels in maternal peripheral blood

^b^ Lipid levels in cord blood. *P*: Significance between preterm group and term group

### Lipid levels in the maternal and cord blood

**Fig 1:**
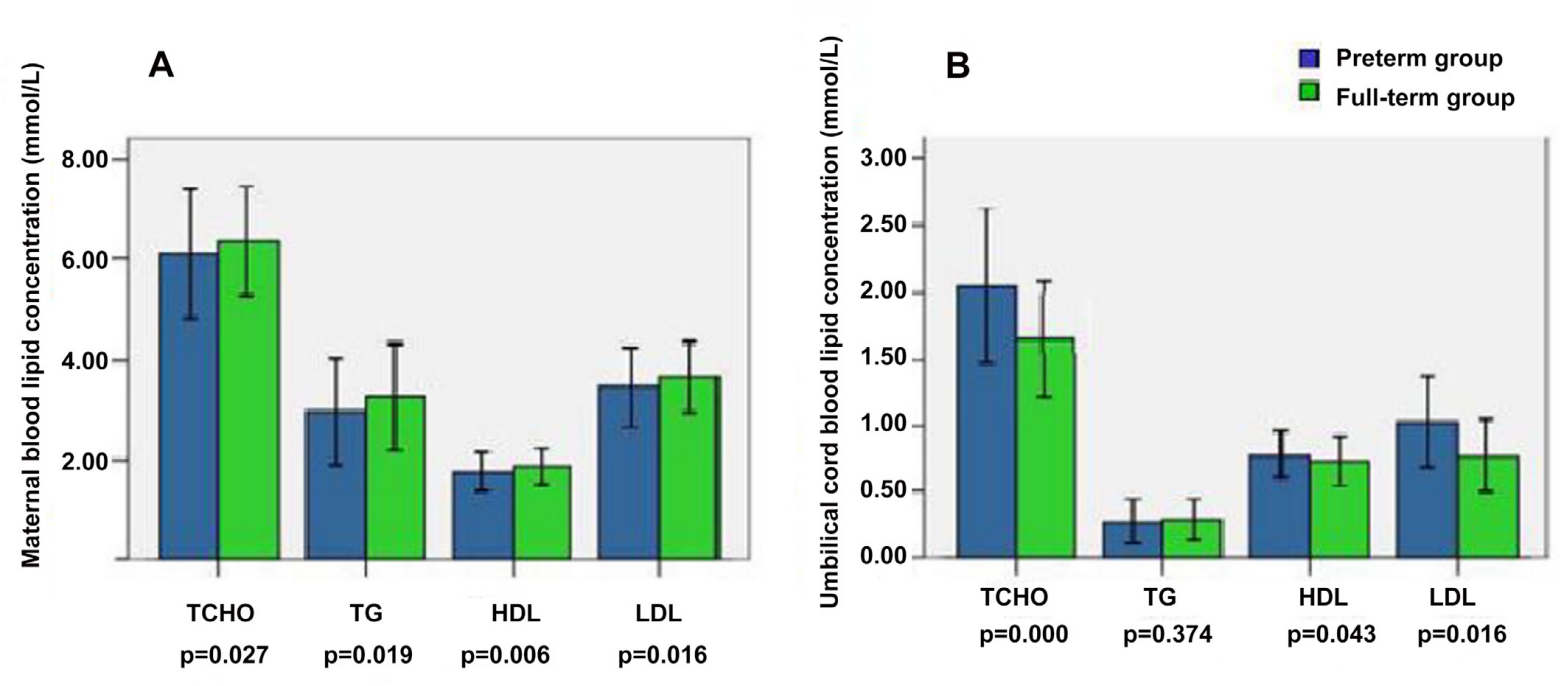
The level of maternal blood and umbilical blood lipids in preterm and full-term groups.

Compared with FTD subjects, the levels of TCHO, TG, HDL, and LDL in the maternal blood were significantly lower while the levels of TCHO, HDL, and LDL in the cord blood were significantly higher in the SPTD subjects (Fig 1). The level of TG in the cord blood was not significantly different between the two groups (Fig 1).

### The genotypes of *ABCA1*, *APOE*, and *HMGCR* genes and allele frequency distribution in maternal and cord blood

*HMGCR* gene rs12916 locus has three genotypes: CC, CT, and TT. The three genotypes and allele frequency distribution were significantly different in the maternal blood cells between the two groups respectively (χ^*2*^ = 6.398, *P* = 0.041), while other genes genotypes and allele frequency distribution in the maternal blood cells were not significantly different (*P* >0.05). Moreover, there were no significant differences in the three genes genotypes and allele frequency distribution in the cord blood cells between the SPTD and FTD groups (*P* >0.05) (Table 3).

**Table 3:** *ABCA1*, *APOE* and *HMGCR* genes polymorphism and variant in maternal blood and umbilical cord blood in full-term and PTBs groups. The figure in the bracket means allele frequency rate.

Polymorphism and variant	Maternal blood in preterm group	Maternal blood in full-term group	Umbilical cord blood in preterm group	Umbilical cord blood in full-term group
***ABCA1***(rs2422493)				
CC	35(0.21)	37(0.24)	34(0.19)	13(0.14)
CT	74(0.44)	64(0.42)	91(0.52)	54(0.57)
TT	58(0.35)	51(0.35)	51(0.29)	28(0.30)
Total	167	152	176	95
Allele frequency C/T	0.43/0.57	0.45/0.55	0.45/0.55	0.42/0.58
***APOE***(rs27412)				
CC	135(0.82)	129(0.85)	146(0.86)	80(0.85)
CT	28(0.17)	22(0.14)	21(0.12)	14(0.15)
TT	1(0.01)	1(0.01)	4(0.02)	0(0%)
Total	164	152	171	94
Allele frequency C/T	0.91/0.09	0.92/0.08	0.92/0.08	0.93/0.07
***HMGCR***(rs12916)				
CC	41(0.24)*	22(0.14)*	40(0.23)	16(0.17)
CT	66(0.39)*	77(0.51)*	82(0.47)	51(0.54)
TT	62(0.37)*	53(0.35)*	54(0.31)	28(0.30)
Total	169	152	176	95
Allele frequency C/T	0.44/0.56*	0.40/0.60*	0.46/0.54	0.44/0.56

* *P* <0.05

### The association between lipid levels and different genotypes of *ABCA1*, *APOE*, and *HMGCR* genes in the maternal and cord blood

In the SPTD group, the TG level in the maternal blood was associated with the different genotypes of the *HMGCR* gene rs12916 loci CC, CT, and TT (*P* = 0.007). The TT group showed the highest TG level, which was significantly higher than that in the CC(*P* = 0.043) and CT groups(*P* = 0.002). The TG level was not significantly different between the CC and CT groups (Table 3). Moreover, the LDL level was associated with the different genotypes of the *HMGCR* gene rs12916 loci CC, CT, and TT in the maternal blood in the SPTD group (*P* = 0.041). Through pairwise comparisons, the TT subjects showed the highest LDL level, which was significantly higher than that in the CC and CT groups (*P* = 0.042, *P* = 0.033). There was no significant difference in the LDL level between the CC and CT groups. The lipid levels in the subjects were not associated with different genotypes of the *HMGCR* gene rs12916 loci in the cord blood. Moreover, the lipid levels were not associated with different genotypes of *ABCA1* gene rs2422493 and *APOE* genes rs7412 determined from the maternal and cord blood of all subjects (Tables 4 and 5).

**Table 4:** The level of maternal blood lipids in different SNPs of *ABCA1* rs2422493, *APOE* rs7412 and *HMGCR* rs12916($\bar{x}\;\pm \;s$, mmol/L).

Lipids	*ABCA1* rs2422493 genotypes			*APOE* rs7412 genotypes			*HMGCR* rs12916 genotypes		
Preterm group	CC (n = 34)	CT (n = 71)	TT (n = 55)	CC (n = 129)	CT (n = 27)	TT (n = 1)	CC (n = 37)	CT (n = 66)	TT (n = 57)
TCHO	6.20±1.50	5.97±1.13	6.40±1.42	6.15±1.30	6.22±1.40	5.29	6.12±1.32	6.01±1.25	6.37±1.41
TG	3.06±1.24	2.93±1.11	3.29±1.25	3.02±1.12	3.29±1.38	4.4	2.95±0.93*	2.80±0.91*	3.44±1.46*
HDL	1.69±0.34	1.78±0.37	1.82±0.46	1.74±0.41	1.89±0.37	1.27	1.83±0.41	1.74±0.42	1.77±0.38
LDL	3.47±0.84	3.38±0.70	3.64±0.89	3.49±0.80	3.50±0.76	2.97	3.37±0.75*	3.41±0.79*	3.71±0.79*
Full-term goup	CC(n = 36)	CT(n = 61)	TT(n = 50)	CC(n = 124)	CT(n = 22)	TT(n = 1)	CC(n = 23)	CT(n = 72)	TT(n = 52)
TCHO	6.42±1.03	6.28±1.25	6.58±1.14	6.41±1.13	6.39±1.40	5.69	6.05±0.99	6.41±1.18	6.56±1.21
TG	3.31±1.52	3.26±1.10	3.51±1.18	3.31±1.18	3.57±1.29	3.8	3.05±0.97	3.48±1.35	3.31±1.18
HDL	1.90±0.39	1.86±0.34	1.92±0.40	1.85±0.37	2.04±0.36	1.86	1.85±0.38	1.87±0.37	1.93±0.38
LDL	3.65±0.68	3.59±0.80	3.78±0.74	3.66±0.73	3.69±0.87	3.08	3.45±0.66	3.66±0.77	3.78±0.76

* *P*<0.05

**Table 5:** The level of umbilical cord blood lipids in different SNPs of *ABCA1* rs2422493, *APOE* rs7412 and *HMGCR* rs12916($\bar{x}\;\pm \;s$, mmol/L). There is no significant difference in lipid level between different genotypes of ***HMGCR*** rs12916 and ***ABCA1*** rs2422493 and ***APOE*** rs7412 in cord blood.

Lipids	*ABCA1* rs2422493 genotypes			*APOE* rs7412 genotypes			*HMGCR* rs12916 genotypes		
Preterm group	CC (n = 32)	CT (n = 71)	TT (n = 42)	CC (n = 122)	CT (n = 16)	TT (n = 4)	CC (n = 33)	CT (n = 71)	TT (n = 43)
TCHO	1.95±0.53	2.07±0.59	2.04±0.54	2.05±0.58	1.88±0.46	1.69±0.39	2.06±0.47	2.02±0.56	2.04±0.63
TG	0.28±0.17	0.25±0.13	0.33±0.19	0.28±0.16	0.22±0.11	0.32±0.19	0.29±0.17	0.27±0.15	0.30±0.17
HDL	0.77±0.16	0.78±0.17	0.76±0.17	0.76±0.17	0.94±0.28	0.74±0.10	0.78±0.18	0.77±0.17	0.77±0.16
LDL	0.97±0.32	1.04±0.35	1.00±0.29	1.02±0.33	0.82±0.28	0.81±0.20	1.02±0.28	1.00±0.32	1.03±0.37
Full-term goup	CC(n = 10)	CT(n = 49)	TT(n = 25)	CCn = 72)	CT(n = 12)	TT(n = 0)	CC(n = 15)	CT(n = 44)	TT(n = 26)
TCHO	1.62±0.50	1.66±0.50	1.69±0.35	1.68±0.46	1.49±0.35		1.69±0.46	1.61±0.46	1.73±0.43
TG	0.27±0.13	0.29±0.15	0.30±0.14	0.29±0.13	0.31±0.17		0.28±0.12	0.31±0.15	0.26±0.13
HDL	0.73±0.22	0.73±0.18	0.76±0.14	0.73±0.18	0.74±0.15		0.80±0.18	0.71±0.18	0.75±0.16
LDL	0.76±0.27	0.78±0.33	0.79±0.20	0.79±0.30	0.66±0.18		0.81±0.29	0.73±0.27	0.83±0.30

## Discussion

This study demonstrated that the lipid profiles varied greatly in maternal and cord blood in two groups. Women had lower concentrations of TCHO, TG, HDL and LDL in maternal blood in SPTD group than in FTD group with gestational age-matched, while had higher concentrations of TCHO, HDL and LDL in cord blood in SPTD group than in FTD group. The results of the lipid profiles in this study were different from the previous studies. Janet et al. [12] reported that high cholesterol and triglycerides at <15 weeks were associated with an increased risk for preterm birth. Lanay et al. [13] reported that extremely low TC, HDLc, and LDLc were also associated with a modest increased risk of PTD, whereas high TC, LDLc and TG increased the risk of spontaneous PTD. In a recent cohort study [14], TCHO level in the first trimester and its change between trimesters were used to predict preterm birth. However, Robin et al.[15] found that lower serum cholesterol was strongly associated with PTD in white mothers. The different results mentioned in the above showed that the disorder of lipid metabolism, whether too high or too low level of lipids, may contribute to the occurrence of the PTD. We also emphasized that in this study the subjects were recruited strictly to minimize the effect of other factors on the level of lipids such as infection, medically indication and cervical incompetence, which were not illustrated clearly on subtype of PTD in most of the literatures.

In a prospective longitudinal study of Turgay' group [16], the levels of TG, TCHO, HDL, LDL increased significantly as pregnancy progressed and lower level of TG was the high risk factors of preterm labor. In order to avoid the bias from the different gestational week we selected the FTD subjects whose gestational age matched to SPTD in our study. More interestingly, the findings of lipid level and SPTD case control status associations in this study were opposite in direction, between maternal peripheral blood and umbilical blood. This result indicated that the fetal lipid metabolism differed remarkably from the maternal and may play a key role in development of SPTD. In Pecks’ study [8], they had provided the evidence for a difference of sera from preterm and term neonates in the ability to promote the reverse cholesterol transport (RCT).

The pathogenesis of abnormal lipid metabolism for SPTD is not clear. Lipoproteins participate in the development of atherosclerosis. An increased cholesterol influx and the oxidation of LDL to oxLDL particles are prerequisites for rapid accumulation of LDL in the macrophages in the vascular wall, leading to foam cell formation and fatty plaque development. Atherosclerotic-like lesions with cholesterol-laden macrophages in the maternal spiral arteries have been found in pathological specimens from pregnancies complicated by preeclampsia, IUGR and PTD [17].

The lipid metabolism between the mother and fetus requires specific receptors (such as VLDL, LDL, HDL, scavenger receptors and LDL receptor-binding protein, etc.) and many placental lipases (such as lipoprotein lipase, phospholipase A2, and intracellular lipase) [18]. Michael et al [19]. reported that high levels of HDL and apolipoprotein A1 were found in the mothers of small for gestational age (SGA) cases, suggesting defective placental transport of HDL. The placenta also seemed to play a important role in the interaction of maternal-fetus lipid metabolism.

Recently, some researchers have investigated the etiologies regarding genetic susceptibility and gene-environment interactions in PTD patients [20,21]. Thus, We hypothesized that the variation of the genes involved in lipid metabolism regulated the lipases expression in the tissue of the mother, fetus, and placental, resulting the different level of lipid profiles between the maternal peripheral and cord blood. Here, we investigate the association between the lipid levels and gene polymorphisms of three major genes *ABCA1* (rs2422493), *APOE* (rs7412) and *HMGCR* (rs12916) involved in cholesterol metabolism in Chinese pregnant women with SPTD. Liu et al [9]. reported that 477C/T SNP in the *ABCA1* gene promoter region exists in the normal Chinese Han population. The HDL-C level in the population with a TT genotype was significantly lower than that in the population with a CC genotype. Here, we observed that the lipid levels in the maternal blood and cord blood were not associated with SNP of 477C/T in the *ABCA1* gene promoter region. The three subtypes (E2, E3 and E4) of *APOE* gene are determined by rs429358 and rs7412 [10]. Steffen et al [20]. observed that the polymorphism of *APOE* gene rs7412 loci had a relationship with PTD. However, our results showed that *APOE* gene rs7412 in the maternal blood and cord blood was not associated with CC, CT, or TT genotypes and the allele frequency distribution was not significantly different between the SPTD and FTD subjects. The polymorphism loci of the *HMGCR* gene rs12916 is located in the non-coding region of *HMGCR* gene 3' UTR, but it may associate with other functional polymorphic loci and affect *HMGCR* gene expression, and thus regulate changes in serum lipoprotein levels. Some researchers [22,23] have found that polymorphism of the HMGCR gene rs12916 loci affected serum TCHO, and LDL levels. Other researchers found the HMGCR gene expressed in placenta for fetal-placental lipid metabolism was different between women with type 1 diabetes (T1DM) and women with no complications [24]. Our results showed that the polymorphisms of the *HMGCR* gene rs12916 loci were associated with changes in the TG and LDL levels in the maternal blood of SPTD subjects, but this association was not found in the umbilical cord blood. Further research are needed to reveal the expression of *HMGCR* gene in the tissue of the mother, fetus, and placental on the pathway of SPTD. Gene polymorphisms of the *APOE* gene rs7412 and the *ABCA1* gene rs2422493 showed neither a correlation with preterm nor term delivery in this study, which was inconsistent with the literatures. The different results may be explained by special status in conception or non-conception, inadequate samples, different population and coordinated action of multiple genes related to the onset and development of preterm. In addition, this study was designed to set the genome of mother and fetus as research objects, the effect of the fathers’ genome on preterm was not considered. Thus, a large sample size is needed to appropriately evaluate the effect of these genetic variants on SPTD.

In summary, compared with FTD subjects, the levels of TCHO, TG, HDL and LDL in the maternal blood were decreased while the levels of TCHO, HDL and LDL in the umbilical cord blood were increased in the SPTD subjects. Moreover, the levels of TG and LDL in the maternal blood were associated with different genotypes of the *HMGCR* gene rs12916 loci, suggesting that *HMGCR* may be a susceptible gene for preterm birth. The findings of lipid level in SPTD cases were opposite in direction between maternal peripheral blood and umbilical blood, which also indicated that the HMGCR gene would express differently in placenta between SPTD and FTD, Further research was needed to give evidences.
